# Supplementary material for: The Systems Biology Research Tool: evolvable open-source software
Source: BMC Syst Biol. 2008 Jun 29;2:55. doi: 10.1186/1752-0509-2-55 (PMC2446383; doi:10.1186/1752-0509-2-55)
Supplement: Additional file 1 — SBRT Archive. An archive of the current version of the Systems Biology Research Tool. [file 1752-0509-2-55-S1.zip › sbrt-1.4.0/doc/developers_guide/api/sbrt/shell/mng/ManagerInput.html]

ManagerInput


|  |  |  |
| --- | --- | --- |
| **Overview** \| **Package** \| **Class** \| **Use** \| **Tree** \| **Deprecated** \| **Index** \| **Help** | |  |
| **PREV CLASS**   **NEXT CLASS** | **FRAMES**    **NO FRAMES**     **All Classes** |  |
| SUMMARY: NESTED \| FIELD \| CONSTR \| METHOD | DETAIL: FIELD \| CONSTR \| METHOD |  |


---


## sbrt.shell.mng Interface ManagerInput<K,V>

**Type Parameters:**: `K` - the key type.: `V` - the value type.

**All Known Implementing Classes:**: MapManagerInput, ProcessInputFile

---

``` public interface ManagerInput<K,V> ```

This interface is used to represent the input to
`KernelProcessManager` objects. A `ManagerInput`
should contain all the information necessary to construct
a process.

**Author:**
:   This interface was written and documented by
    Jeremiah Wright while in the Wagner lab.

---

| **Method Summary** | |
| --- | --- |
| `java.lang.String` | `getName()`             Returns the name of this manager input. |
| `V` | `getOptionalValue(java.lang.Object key)`             Returns the value corresponding to the provided key. |
| `V` | `getRequiredValue(java.lang.Object key)`             Returns the value corresponding to the provided key. |
| `boolean` | `hasValueFor(java.lang.Object key)`             Indicates if a value exists for the provided key in this manager input. |
| `java.util.Map<K,V>` | `toMap()`             Returns the information contained in this input as a map. |

**Method Detail**

### hasValueFor

```
boolean hasValueFor(java.lang.Object key)
```

:   Indicates if a value exists for the provided key
    in this manager input.

    :   **Parameters:**: `key` - the key whose presence in this input is to be tested. **Returns:**: `true` if this input contains a value for the specified key.

---


### getRequiredValue

```
V getRequiredValue(java.lang.Object key)
```

:   Returns the value corresponding to the provided key.

    :   **Parameters:**: `key` - the key whose value is to be returned. **Returns:**: the value corresponding to the provided key. **Throws:**: `ApplicationException` - if the value does not exist.

---


### getOptionalValue

```
V getOptionalValue(java.lang.Object key)
```

:   Returns the value corresponding to the provided key.

    :   **Parameters:**: `key` - the key whose value is to be returned. **Returns:**: the value corresponding to the provided key, or `null` if the value does not exist.

---


### toMap

```
java.util.Map<K,V> toMap()
```

:   Returns the information contained in this input as a map.

    :   **Returns:**: keys mapped to their respective values.

---


### getName

```
java.lang.String getName()
```

:   Returns the name of this manager input.

    :   **Returns:**: the name of this manager input.


---


|  |  |  |
| --- | --- | --- |
| **Overview** \| **Package** \| **Class** \| **Use** \| **Tree** \| **Deprecated** \| **Index** \| **Help** | |  |
| **PREV CLASS**   **NEXT CLASS** | **FRAMES**    **NO FRAMES**     **All Classes** |  |
| SUMMARY: NESTED \| FIELD \| CONSTR \| METHOD | DETAIL: FIELD \| CONSTR \| METHOD |  |


---
